# Evaluation of potential circulating biomarkers for prediction of response to chemoradiation in patients with glioblastoma

**DOI:** 10.1007/s11060-016-2178-x

**Published:** 2016-07-21

**Authors:** Myra E. van Linde, Johannes C. van der Mijn, Thang V. Pham, Jaco C. Knol, Laurine E. Wedekind, Koos E. Hovinga, Esther Sanchez Aliaga, Jan Buter, Connie R. Jimenez, Jaap C. Reijneveld, Henk M. W. Verheul

**Affiliations:** 1 Department of Medical Oncology, VU University Medical Center, Amsterdam, The Netherlands; 2 Department of Neurosurgery, Neuro-oncology Research Group, VU University Medical Center, Amsterdam, The Netherlands; 3 Department of Neurosurgery, VU University Medical Center, Amsterdam, The Netherlands; 4 Department of Radiology, VU University Medical Center, Amsterdam, The Netherlands; 5 Department of Neurology, VU University Medical Center, Amsterdam, The Netherlands

**Keywords:** Glioblastoma multiforme, Biomarker, Proteomics, First-line treatment

## Abstract

Surgery followed by chemoradiation and adjuvant chemotherapy is standard of care for patients with a glioblastoma (GBM). Due to its limited benefit, an upfront method to predict dismal outcome would prevent unnecessary toxic treatment. We searched for a predictive blood derived biomarker in a cohort of 55 patients with GBM. Increasing age (HR 1.03, 95 % CI 1.01–1.06), and postoperative tumor residue (HR 1.07, 95 % CI 1.02–1.15) were independently associated with unfavourable progression free survival (PFS) in these patients. Corticosteroid use before start of chemoradiaton was strongly predictive for outcome (HR 3.26, 95 % CI 1.67–6.39) with a mean PFS and OS in patients using corticosteroids of 7.3 and 14.6 months, versus 16.1 and 21.6 months in patients not using corticosteroids (p = 0.0005, p < 0.0067 respectively). Despite earlier reports, blood concentrations of YKL-40, Fetuin-a and haptoglobin were not predictive for response. In addition, serum peptide profiles, determined by MALDI-TOF mass spectroscopy, were not predictive as well. In conclusion, further biomarker discovery studies are needed to predict treatment outcome for patients with GBM in the near future.

**Electronic supplementary material:**

The online version of this article (doi:10.1007/s11060-016-2178-x) contains supplementary material, which is available to authorized users.

## Introduction

Despite intensive treatment, general prognosis of patients with a glioblastoma multiforme (GBM) remains dismal with a median survival rate of approximately 15 months and a 2 year survival rate of around 25 % [1]. Current standard first-line treatment takes about 9 months to be completed and consists of surgical debulking, followed by radiotherapy with concomitant and adjuvant temozolomide [1]. This treatment regimen induces a significant burden due to its toxicity (e.g. bone marrow toxicity and fatigue), and takes a relatively long period of time. Upfront selection of GBM patients who will have no benefit from this multimodality treatment, is currently not possible. The methylation of the MGMT-gene promotor was shown to be a prognostic and a relatively weak predictive biomarker for the response to temozolomide monotherapy, and therefore clinically used only for older frail patients, when tolerability of the combination treatment is questionable [2]. IDH1 mutations were postulated as a potential predictive biomarker, but its value has to be confirmed before implementation in daily practice [2, 3]. Assessments of both these markers and other potential genetic biomarkers require tumor tissues, which is not available at any given time during the treatment or follow up. Tumor tissue from patients with GBM is more difficult to obtain than a venous puncture. With a blood derived tumor marker consecutive follow-up evaluations of treatment response would be possible in order to prevent unnecessary treatment or treatment related toxicity. The availability of a blood biomarker, obtained through an easy venous puncture, predicting response to treatment in an early stage, would be an important advantage for GBM patients. It is known that proteolytic cascades within the tumor microenvironment may generate disease specific proteins and peptide fragments that are being released in blood of patients. Also proteases themselves are shed by tumors, inducing modulations of blood proteins while in the circulation as well as after blood collection ex vivo [4, 5]. Therefore serum and plasma have been studied as a biomarker source.

In previous studies it was found that the YKL-40 concentration in serum and the YKL-40 expression in tumors is elevated [6, 7]. Comparative genomic hybridization studies and gene expression array analysis of GBM tumor tissue revealed that YKL-40 was associated with chromosome-10 loss and poor clinical outcome [8]. Fetuin-A is one of the most abundant plasma glycoproteins and was also identified as a potential biomarker for patients with a high grade glioma. The serum Fetuin-A concentration, before start of treatment, was associated with survival in patients with GBM, especially when combined with age and Karnofsky performance score (KPS) [9]. Similarly, haptoglobin was found to be significantly up-regulated in serum of GBM patients and may play a role in tumor angiogenesis and proliferation of cancer cells [10]. A correlation between decreased platelet counts during concurrent radiotherapy and temozolomide treatment and a positive effect on overall survival (OS) has been described in patients with a GBM [11].

In this exploratory study, we investigated whether serum peptides as well as serum concentrations of haptoglobin, thrombocytes, YKL-40 and Fetuin-a, could predict treatment response in newly diagnosed GBM patients.

## Materials and methods

### Patient population

This study was conducted in the VUmc and was registered by the Medical Ethical Committee (2004/139). Each patient provided written informed consent. Included patients with histologically confirmed newly diagnosed glioblastoma received standard first-line treatment [surgery followed by irradiation (total of 60 Gy), with concomitant temozolomide (75 mg/m^2^) and adjuvant six cycles of temozolomide, dosed 150–200 mg/m^2^, 5 days per week in cycles of 4 weeks]. Serum samples were collected at three different time points: postoperative, after chemoradiation, and after completion of the adjuvant treatment phase.

Postoperative tumor volume or tumor residue was calculated by using iPlan Net3.0.0 software (BrainLAB AG, Germany) by subtracting T1 spontaneous hyperintense signal on MRI (due to blood degradation products) in the resection cavity from the contrast enhancing residue.

Disease progression was assessed using the Macdonald criteria [12].

### Serum sample handling

Venous blood was collected in 7 mL tubes (Cat #367615,BD, Franklin Lakes, NJ) and allowed to clot for 30 min-1 h at RT. The 1 h at RT is an optimal time window that is needed to allow endogenous cancer specific proteases to cleave abundant blood proteins released by the clotting process, described by Villanueva et al. [5]. Blood was centrifuged at 1500*g* for 10 min at RT. Serum aliquots of 300  µL were taken, frozen and stored in polypropylene Eppendorf tubes at −80 °C until further use. All samples underwent one freeze thaw cycle before measurement. To keep pre-analytical variables as constant as possible in order to maintain the quality of the samples, a standardized, reproducible workflow has been implemented in our translational proteomic facility as described before [14].

### Sample spotting and MALDI-TOF mass spectrometry

Peptide capture and measurement was performed as described before [13]. MALDI-TOF-MS was performed in reflectron positive mode on the 4800 MALDI-TOF/TOF mass spectrometer (Applied Biosystems) with 5000 shots per spectrum. The instrument was calibrated using a calibrant peptide mixture. Linear mode spectra were acquired from *m/z*800 through 4000.

### Serum protein measurements

Protein concentrations of previously published candidate biomarkers were measured by ELISA. YKL-40 (Sunred Biological Technology Co) and Fetuin-A (α2-Heremans-Schmid glycoprotein, R&D systems) measurements were performed according to manufacturer’s instructions. Haptoglobin concentrations were determined by standard immunonefolometry, using a immage 800 immunochemistry system (Beckman Coulter Inc.). Thrombocyte counts were measured on a CD4000 impedance hematology analyzer.

### Statistical analysis

Treatment outcome was determined by calculating time between surgery and disease progression [progression free survival (PFS) or death (OS)]. Kaplan–Meier graphs, histograms of treatment response and non-linear fitting of Gaussian distributions were performed in Graphpad prism (version5). Concentrations of candidate protein biomarkers were determined after log-transformation of absorbance values. Data was imported in SPSS (version20). Log transformation was applied to reach normal distributions. Pearson’s correlation coefficient was determined to assess linear relationship between parameters. Serum peptide spectra were analyzed using our OPL-Analyzer software package as described before [14]. The data was pre-analyzed by preparation of metadata and the processing of raw mass spectrometry signals, which consists of peak detection, alignment, normalization and deisotoping. The peptides were further subjected to intensity filtering, requiring the median intensity of at least one group to be greater than 80 units and the fold change of the median intensities of two groups greater than 1.5. Candidate peaks were examined visually by spectra overlay in Matlab and subjected to principle component analysis (PCA), unsupervised cluster analysis and univariate Cox regressions. All these analysis were performed without assignment of response group labels. Predictive signatures were generated by support vector machine (SVM), random forests (RF) and *k*-nearest neighbor statistical models, using peptide profiles from 24 randomly selected patients as training set. Performance of the predictive signatures was tested in the 26 remaining patients as independent test-set. Multivariate Cox regressions and subgroup analysis were performed to compare short versus long responders as determined by PFS. To view distribution of the treatment response a histogram was generated from PFS and a Gaussian distribution was fitted. The goodness of fit and 95 % confidence intervals will be assessed in Graphpad prism. For the subgroup analysis the two tailed unpaired Student’s *t* test was used. All p values smaller than 0.05 were considered statistically significant.

## Results

### Patient cohort and treatment response

Between 2005 and 2012, 55 patients, were included. Table 1 summarizes the patient characteristics.

**Table 1:** Patient characteristics

Characteristic	PFS <16 months	PFS >16 months
Patients (n)	39	16
Age—year		
Median	59*	48*
Range	18–75	22–66
Gender—no (%)		
Female	12 (31)	4 (25)
Male	27 (69)	12 (75)
Histology—no (%)		
Glioblastoma	39 (100)	16 (100)
Tumor location—no (%)		
Left	14 (36)	5 (31)
Right	20 (51)	11 (69)
Multifocal	5 (13)	–
WHO performance		
0	22 (56 %)	8 (50 %)
1	15 (38 %)	6 (38 %)
2	1 (3 %)	
Missing	1 (3 %)	2 (13 %)
Extent of surgery		
Biopsy	4 (10 %)	–
Debulking		
Partial	15 (38 %)	10 (63 %)
Maximal	9 (23 %)	6 (38 %)
Unknown	11 (28 %)	
Time from diagnosis to radiotherapy—weeks		
Median	4.0	4.1
Range	1.7–57.3	3.1–5.7
Radiotherapy—dose		
42 Gy	2 (5 %)	
75 Gy	37 (95 %)	16 (100 %)
TMZ—dose		
0 mg/m^2^	2 (5 %)	–
75 mg/m^2^	37 (95 %)	16 (100 %)
Dose reductions (number of pat)	4 (10 %)	2 (13 %)
Average adjuvant cycles—no	4.2	5
Corticosteroid therapy		
Yes	19 (49 %)**	1 (6 %)**
No	20 (51 %)**	15 (94 %)**
Anti-epileptic therapy		
Yes	18 (46 %)	9 (56 %)
No	20 (51 %)	7 (44 %)
Missing	1 (3 %)	–

*p = 0.02 and **p = 0.002

Two of the 55 patients were treated differently, with chemoradiation at a dose of 42 Gy, without adjuvant temozolomide. The mean number of cycles completed in the adjuvant phase was five (range 1–6). In 12 patients, dose reductions with temozolomide were needed in the adjuvant phase, because of toxicity CTC grade 3 and 4.

**Fig. 1:**
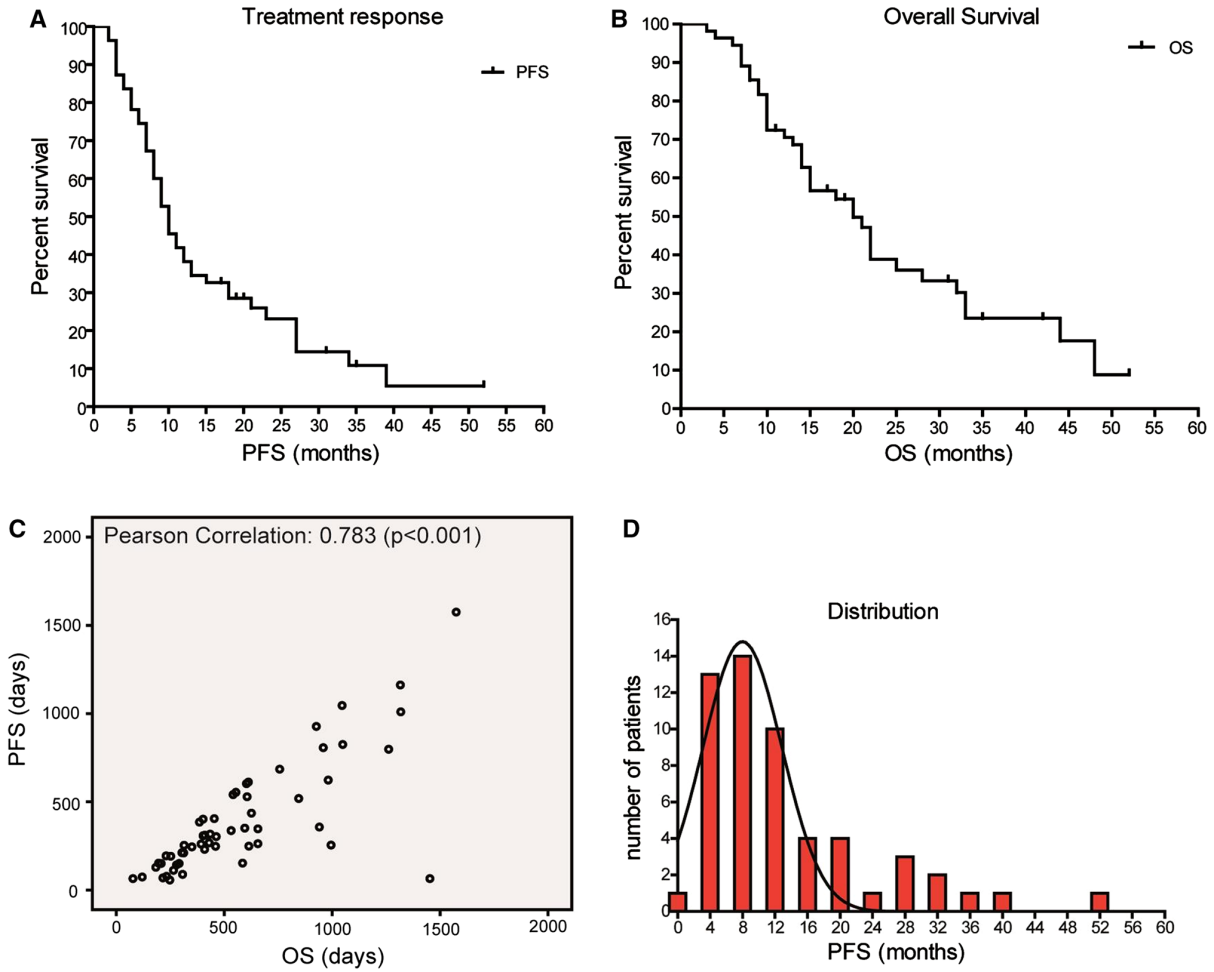
**a** Outcome of first-line treatment in patients with glioblastoma. Treatment response of patients with glioblastoma that received radiotherapy and concomitant and adjuvant temozolomide. Kaplan–Meier curves show that (**a**) median PFS is 10 months (range 2–52 months). **b** Outcome of first-line treatment in patients with glioblastoma. Treatment response of patients with glioblastoma that received radiotherapy and concomitant and adjuvant temozolomide Kaplan–Meier curves show that (**b**) median OS is 15 months (range 3–52 months). **c** Correlation between PFS and OS by Pearson correlation. Correlation between PFS and OS of patients with glioblastoma who received radiotherapy and concomitant and adjuvant temozolomide was assessed by Pearson correlation. **d** Normal distribution. General response (PFS) tends to follow a normal distribution with a distinct group of patients that responds beyond 16 months

Follow-up of treatment response (PFS) was censored on November 8th 2012 in nine patients. Survival was censored in 18 out of 55 patients. Median PFS was 10 months (range 2–52 months) (Fig. 1a). Median OS was 15 months (range 3–52 months), and 2 year survival was 25 % (Fig. 1b). Correlation between PFS as determined by the Macdonald response criteria and OS was measured. A correlation was present with a Pearson’s correlation coefficient of 0.78 (p < 0.001) (Fig. 1c). To view distribution of the treatment response a histogram was generated from PFS and a Gaussian distribution was fitted. The goodness of fit and 95 % confidence intervals were assessed in Graphpad prism. The observed responses fitted well with a Gaussian distribution model with a R^2^ of 0.86 and narrow confidence interval for the mean (6.6–9.4 months). Mean of the Gaussian distribution was 8.0 months with a standard deviation of 4.9 months (95 % confidence interval 3.5–6.3 months) (Fig. 1d).

### Correlation between patient characteristics and treatment outcome

**Fig. 2:**
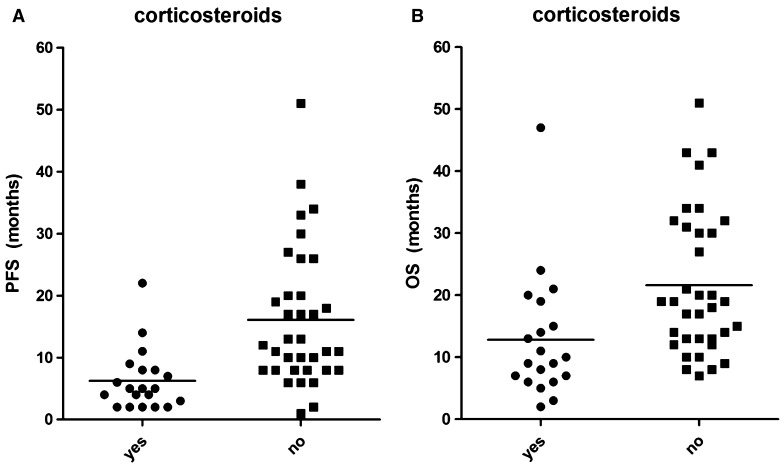
**a** PFS in the group of patients using corticosteroids before start of chemoradiation versus the group of patients not using corticosteroids before start of chemoradiation. A significantly difference has been found between mean PFS of 7.3 months in patients using corticosteroids before start of chemoradiation versus 16.1 months in patients not using corticosteroids before start of chemoradiation (p = 0.0005). **b** OS in the group of patients using corticosteroids before start of chemoradiation versus the group of patients not using corticosteroids before start of chemoradiation. A significantly difference has been found between mean and OS of 14.6 months in patients using corticosteroids before start of chemoradiation versus 21.6 months in patients not using corticosteroids before start of chemoradiation (p = 0.0067)

By using univariate Cox regression analysis, significant correlations between age at the time of surgery and PFS (p = 0.008) between postoperative tumor residue and PFS (p = 0.010), and between use of corticosteroids and PFS (p < 0.001) were found. No correlations were found between treatment response (PFS) and extent of resection, WHO performance score, gender, tumor location, or use of anti-epileptic drugs (Table 1). We reasoned that it would be clinically useful to identify the patients that will have long standing responses. Patients with PFS longer than 16 months (2× standard deviation of the mean) have substantial benefit of treatment and were therefore classified as the favorable response group and as long responders. We compared significantly associated factors in the short (<16 months) versus long (>16 months) responders. Both age and corticosteroids use were significantly different between the two response groups, while calculated postoperative tumor residue was not significantly different between the two response groups (p = 0.30). Multi-variate Cox regression analysis showed that both increasing age (HR 1.03, 95 % CI 1.01–1.06), postoperative tumor residue (HR 1.07, 95 % 1.02–1.15), and corticosteroid use (HR 3.26, 95 % CI 1.67–6.39) before start of chemoradiation (i.e. postoperatively) are independently associated with unfavorable treatment outcome. It is evident that only corticosteroid use before start of chemoradiation was strongly predictive for outcome. We found a significantly difference between mean PFS and OS of 7.3 and 14.6 months in patients using corticosteroids before start of chemoradiation, versus 16.1 and 21.6 months in patients not using corticosteroids before start of chemoradiation (p = 0.0005 and p = 0.0067, respectively) (Fig. 2a, b).

### No correlation between postoperative serum peptide profile and treatment response

From 50 patients, serum was collected before start of chemoradiation. The median time between operation and the first sample taken was 21 days, the mean time was 23 days (range 13–33 days).

**Fig. 3:**
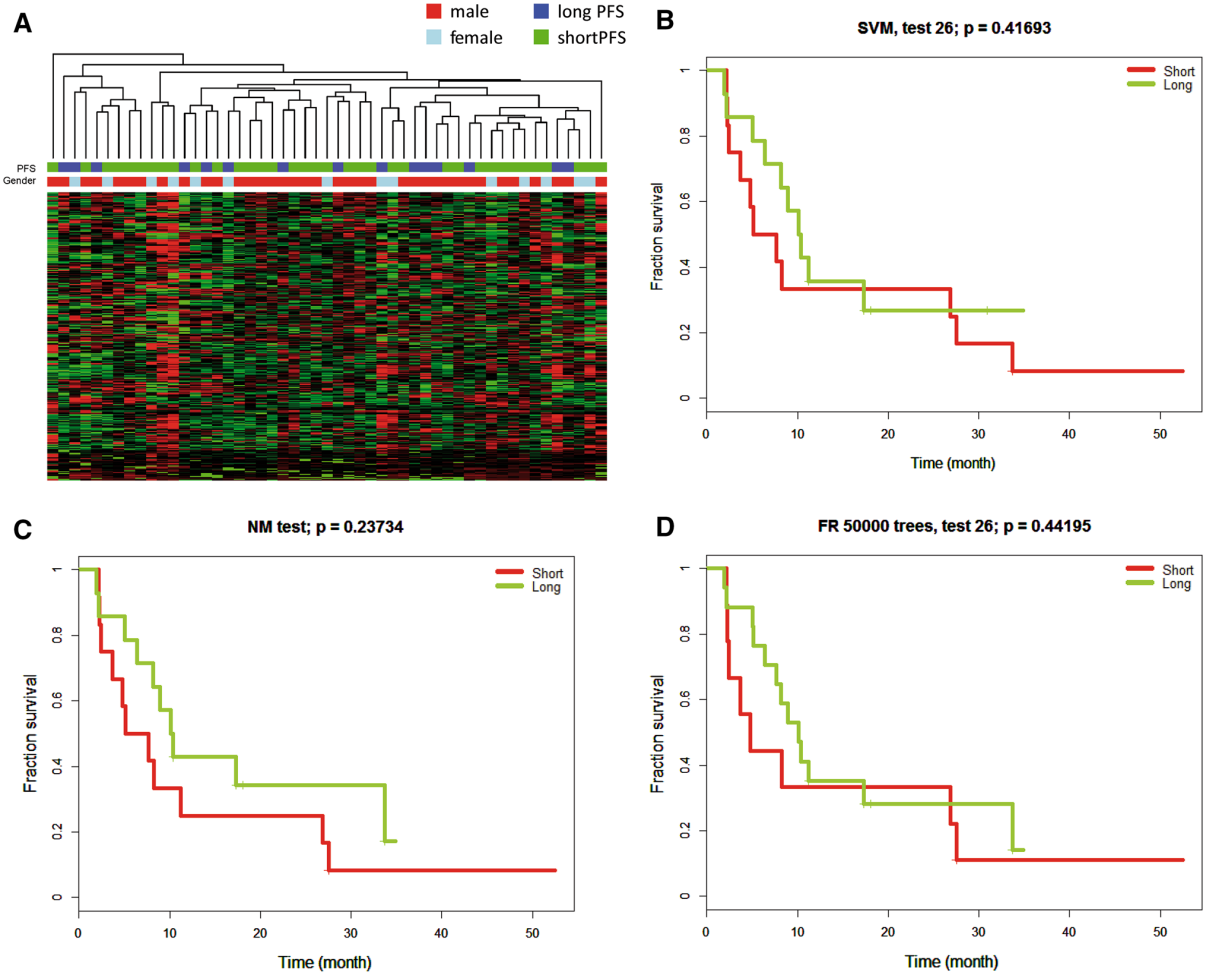
**a** Categorization in a short versus long treatment response group based on serum peptide profiling. Serum was collected postoperatively, before start of chemoradiation and peptides were profiled by MALDI-TOF-MS. Unsupervised cluster analysis was performed and samples were labeled by gender and good (PFS >16 months) versus poor treatment response (PFS <16 months). **b**–**d** Result of testing potential predictive classifiers to predict treatment response. Predictive classifiers were generated from the peptide profiles of 18 patients. Each classifier was tested in an independent patient set of 26 patients. Signatures generated by support vector machine (**a**), *k*-nearest neighbor (**b**) and random forrest (**c**) did not predict treatment response (**b**–**d**)

All serum samples were measured by MALDI-TOF mass spectrometry. After initial quality control and pre-processing of the mass spectrometry chromatograms, in total 274 peptide peaks were detected above the signal intensity threshold. Principal component analysis and unsupervised cluster analysis were performed to look at variation between samples. Both analyses showed three serum samples clustering separately from all other samples. These three serum samples were hemolytic before measurements and spectra were of poor quality and were therefore excluded from further analyses. We performed another unsupervised cluster analysis and labeled samples by gender and poor (<16 months) versus favorable (>16 months) treatment response (Fig. 3a). No grouping was observed by either gender or treatment response. Univariate Cox regression analysis was performed to investigate correlation between peptide levels and treatment response (PFS). In total 36 peptide peaks showed significant correlation with PFS. Visual inspection of the 36 raw peptide spectra was done and revealed 14 peaks of sufficient quality for follow-up. After correction of p-values for multiple testing, none of the peptide peaks was significantly different between the two groups.

Despite these results in univariate analysis, we argued that combination of multiple peptides together may still yield predictive power. Therefore, we used three different statistical models to generate the best performing predictive signatures in a subset of 18 patients. Using support vector machine, a 24-peptide signature was developed for response prediction. Still, the performance of the signature in the independent test-set was insufficient to separate responding from unresponsive patients (p = 0.42) (Fig. 3a). Similar results were obtained with different training strategies such as *k*-nearest neighbour (p = 0.24) and random forests (p = 0.44) (Fig. 3b–d).

### Candidate protein biomarkers in serum samples

**Fig. 4:**
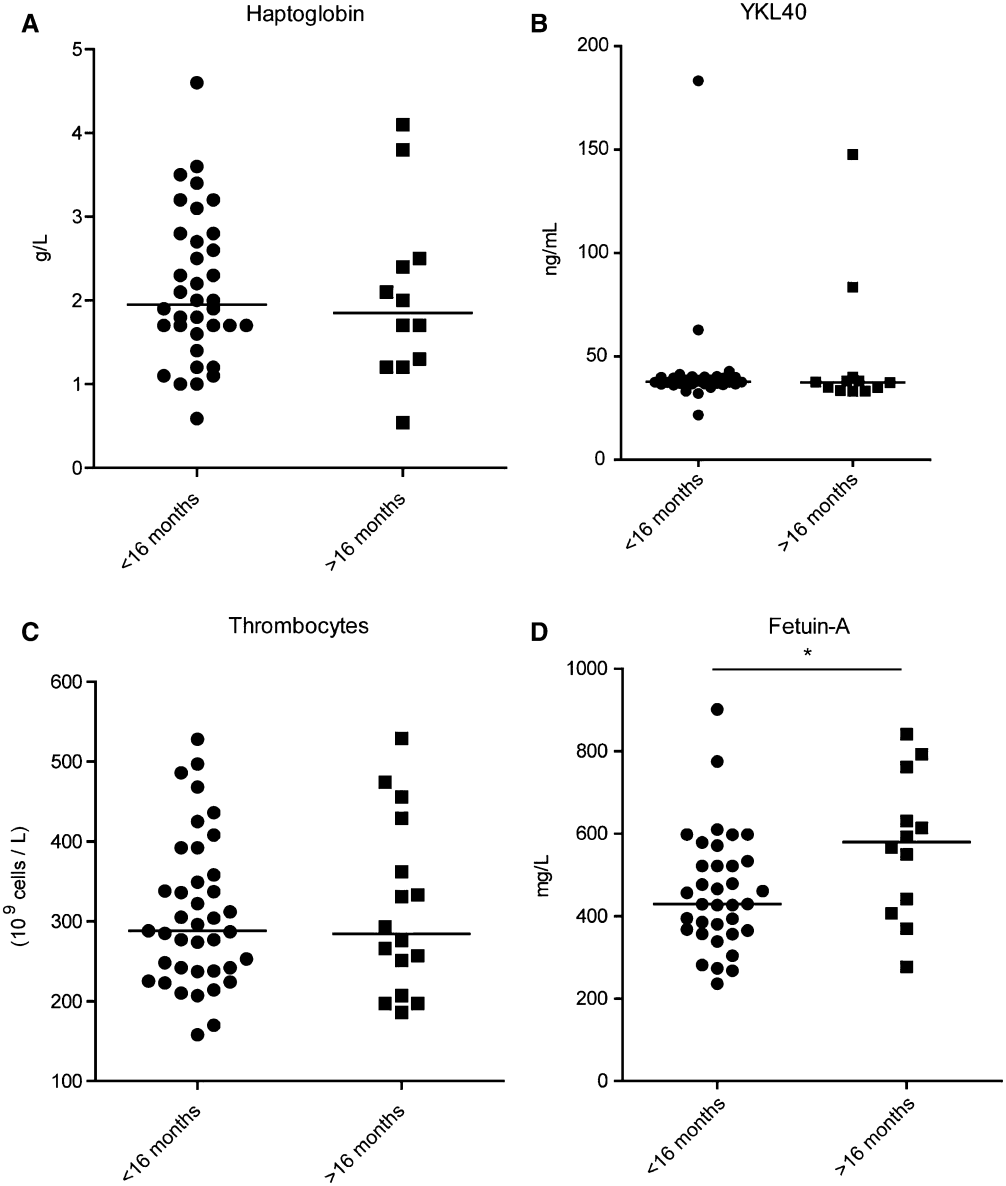
Analysis of candidate biomarkers and correlation of candidate biomarkers with treatment response. Protein concentrations of previously published candidate biomarkers were measured in serum samples by ELISA (**a, d**) or immunonefolometry. **b** Thrombocyte counts were measured by a CD4000 impedance hematology analyzer. **c** Correlation with treatment response was determined by Cox regression (not shown) and group comparison. Fetuin-a protein concentrations were significantly different between good and poor response groups (p = 0.03)

From 47 patients, enough serum was available for measurements of haptoglobin, YKL-40, Fetuin-A and thrombocytes, after initial serum peptide profiling. Median haptoglobin concentration was 1.9 g/L (range 0.5–4.6 g/L), median thrombocyte count was 288 × 10^9^ cells/L (range 158–529 × 10^9^ cells/L). Haptoglobin concentration was above the reference level in 18 patients (38 %), while thrombocytosis was present in 7 patients (13 %). A significant decrease of haptoglobin concentrations was present after completing the adjuvant phase, but not after completing chemoradiation (p < 0.0001, Suppl.Fig.S1). No significant change in thrombocyte counts was found (Suppl.Fig.S2). Similarly, no significant correlation was found between haptoglobin concentrations (p = 0.16) or thrombocyte counts and PFS. Median YKL-40 and Fetuin-A concentrations were 37.8 ng/mL (range 21.8–183.3 ng/mL) and 460.9 mg/L (range 236.4–901.4 mg/L) respectively. Both YKL-40 and Fetuin-A concentrations were significantly increased, when comparing the pre-chemoradiation samples with the samples after chemoradiation (p = 0.0002, p = 0.0026, suppl.Fig.S3, S4). Both YKL-40 and Fetuin-A concentrations were not associated with PFS as determined by Cox regression analysis (p = 0.54 and p = 0.25 respectively). However, when comparing concentrations in our previously identified response groups, Fetuin-A was significantly increased in patients with favorable treatment response (p = 0.03) (Fig. 4a–d).

## Discussion

We performed a population-based study of 55 patients with a newly diagnosed glioblastoma, who were treated with current standard treatment. In comparison to the initial registration trial, our patient cohort is a representative cohort with an OS of 14.6 months and a PFS was 10 months [1]. The response to chemoradiation usually tends to follow a normal distribution, while in this patient cohort a distinct subgroup existed that responded longer than 16 months and survived the first 2 years. Similar to other studies, we found younger age, no corticosteroid treatment before start of chemoradiation and low postoperative tumor volume important predictive factors for a favorable treatment response and survival [3, 19]. In contrast to some other studies, performance status and extent of surgical resection did not predict for treatment outcome.

In general, but for brain tumors in particular, blood represents an attractive platform for prediction and monitoring of treatment response since it is easy to obtain by venous puncture. In previous studies peptide profiles in serum of patients with glioblastoma differed from healthy donors [10, 20]. Gollapalli and colleagues compared the serum proteome of patients with GBM and healthy subjects and revealed 55 differentially expressed and statistically significant (p < 0.05) protein spots [20]. Several studies demonstrated that serum peptide profiles can be used for diagnostic purposes and early detection of cancer [21, 22]. This indicates that peptide profiles represents a sensitive tool, which may be useful to guide therapeutic decisions in daily practice. Moreover, the serum peptidome was shown to harbor robust biomarkers for prediction of treatment response, including chemoradiation, in other types of cancer [23, 24]. We collected serum from GBM patients postoperative, after chemoradiation, and after completion of the whole treatment. Analysis of the serum peptide profiles did not yield a predictive signature, so we concluded that peptides in serum collected after surgery cannot predict outcome of multimodality treatment. This may be due to the fact that GBM is a heterogeneous disease with different molecular abnormalities that may preclude the ability to identify a single set of proteins that reflects outcome. The total number of peptides identified in this analysis was comparable to the total number of identified peptides in a previous study from our department [14]. They found that serum peptidome profiling may aid in prediction of treatment outcome of patients with advanced non-small cell lung cancer (NSCLC) treated with chemotherapy [14]. Furthermore, the clinical predictive potential of peptide profiling for systemic treatment was shown by Gregorc et al. They found a predictive proteomic signature in patients with NSCLC treated with second-line erlotinib or chemotherapy [26] and supported by multiple studies evaluating MS-based serum and plasma peptidome profiling for prediction of treatment outcome in patients with solid malignancies as summarized previously [27]. Our inability to find a predictive peptide profile for GBM patients may indicate that pre-operative serum might be a more suitable source for this analysis. However, even after optimal resection of a GBM, (microscopic) tumor residue is being left. In addition, patients with advanced NSCLC in the study of Voortman and colleagues were treated with chemotherapy only, and they did not receive any surgery [14]. Others reported that serum peptide profiles can also be used as a sensitive measure for early prediction of disease relapse after surgery [28, 29]. In this study, we observed no significant distinction between patients with maximal versus subtotal resection in unsupervised cluster analysis [data not shown], suggesting that peptide profiles may have less potential in GBM patients.

Serum YKL-40 concentration has been suggested as a useful biomarker for monitoring disease relapse in GBM patients [30, 31]. YKL-40 was shown to be overexpressed in tumor tissue of patients, enhancing tumor growth and angiogenesis [34]. We found very little variation in YKL-40 protein concentrations in serum of patients throughout the disease course. This is a remarkable finding, since others even reported a transient decrease of protein levels postoperatively, while using the same bioassay [31]. We did not observe any correlation between extent of resection and serum YKL-40 that was previously reported in GBM patients [35]. We also found no correlation at different time points between postoperative tumor volume and serum YKL-40 concentrations.

In contrast to YKL-40, Fetuin-A and haptoglobin are proteins that are also abundantly present in serum of healthy individuals. Fetuin-A was found to be increased, while haptoglobin was demonstrated to be decreased in GBM patients with favorable prognosis [9, 10]. We observed a similar trend without significance for both molecules in our regression analysis. Significant changes in biomarker concentrations were detected when comparing pre-chemoradiation samples with samples taken after completing whole treatment, indicating that timing of sampling may be of importance. Interestingly, Fetuin-A was significantly increased in pre-treatment samples when comparing the poor and good response groups, suggesting that the power of our analysis may have been insufficient.

Although these biomarkers could still be of value, correlations may be too weak for robust prediction of treatment response. Even, grouping these individual biomarkers in high and low levels did not provide any predictive value (data not shown). All currently established predictive biomarkers are derived from tumor tissue. The advent of large scale genomic profiling allows comprehensive studies of the driver molecules in glioblastoma. For example PDGFR and EGFR were identified as important molecules, together with p53, RB1 and NF1 [36]. Molecular alterations however may change over time, particularly when treatment is involved, urging repeated surgical interventions. The methylation of the MGMT-gene promotor and IDH1 mutations as prognostic and/or predictive factors were not evaluated in our study due to its retrospective nature. Most probably their potential value will not change our inability to find a reliable prognostic or predictive circulating biomarker in serum for patients with GBM.

Still, the non-invasive platforms in blood that are currently in development may become part of future patient management. Circulating tumor cells or extracellular vesicles are shed by tumors into the circulation and may harbor tumor specific molecules [37]. Epigenetic features such as MGMT promoter methylation status are strong predictors of treatment response and may be assessed in serum [38]. In addition, we previously found that platelet contents may provide a biomarker for disease and treatment response evaluations [39]. We have demonstrated that tumor cells transfer (mutant) RNA into blood platelets in vitro and in vivo, and showed that blood platelets isolated from glioma and prostate cancer patients contain the cancer-associated RNA biomarkers EGFRvIII and PCA3, respectively. In addition, gene-expression profiling revealed a distinct RNA signature in platelets from glioma patients compared with normal control subjects [39]. Therefore we are currently sampling platelets from patients with a GBM that undergo first-line treatment. These tools can help monitoring drug resistance, guide therapeutic decisions to improve outcome for patients with GBM.

## Electronic supplementary material

Below is the link to the electronic supplementary material.

Supplementary material 1 (DOCX 263 KB)
