# Supplementary material for: Evaluation of potential circulating biomarkers for prediction of response to chemoradiation in patients with glioblastoma
Source: J Neurooncol. 2016 Jul 21;129:221–30. doi: 10.1007/s11060-016-2178-x (PMC4992035; doi:10.1007/s11060-016-2178-x)
Supplement: Supplementary file 1 — Supplementary material 1 (DOCX 263 KB) [file 11060_2016_2178_MOESM1_ESM.docx]

**Supplementary Figures**


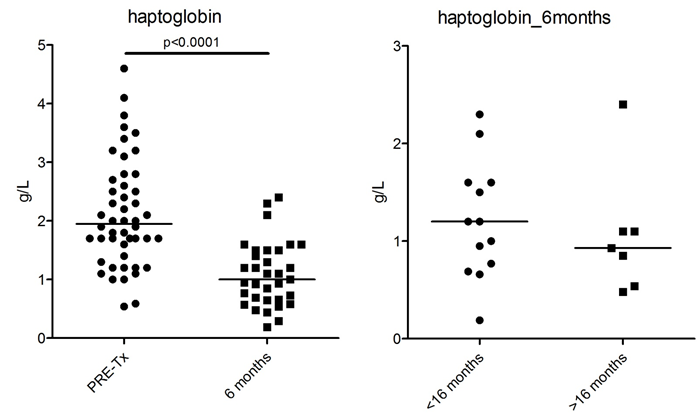


**Suppl. Figure 1│**Haptoglobin level is significantly decreased after 6 months. No difference between favourable (>16 months) versus poor (<16 months) responders.


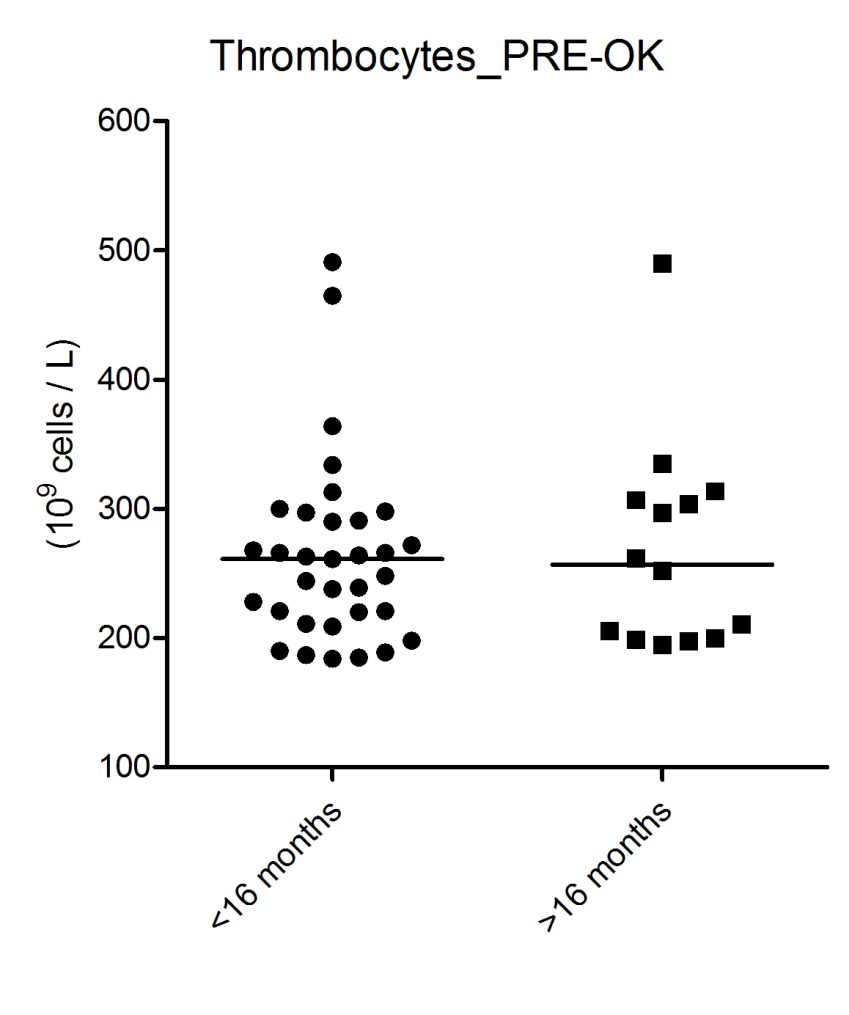


**Suppl. Figure 2│**No difference in pre-surgery platelet counts between favourable (>16 months) vs poor


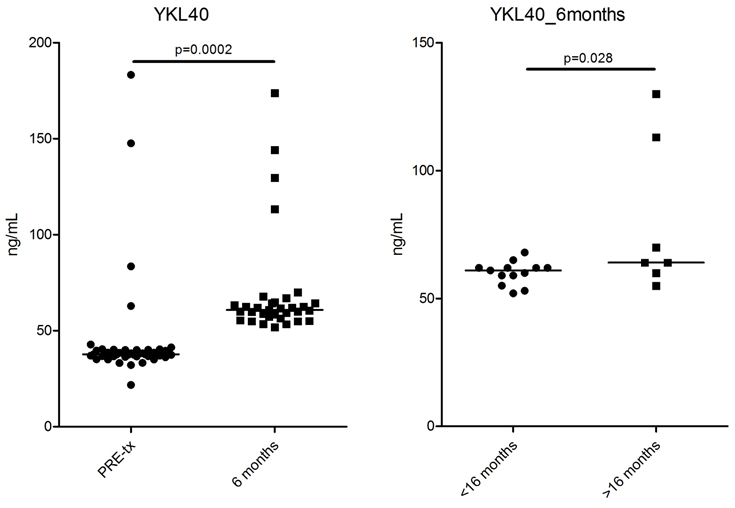


**Suppl. Figure 3│**YKL40 level is significantly increased after 6 months. YKL40 is significantly increased in patients with prolonged response after 6 months.


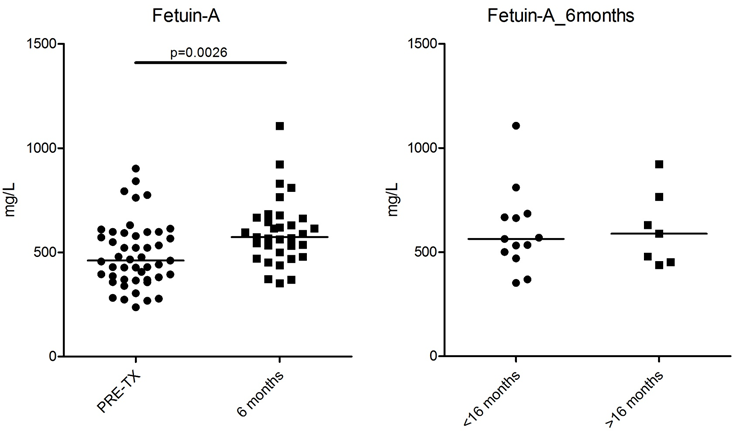


**Suppl. Figure 4│**Fetuin A is significantly increased after 6 months. No difference between favourable (>16 months) versus short (<16 months) responders.
